# Suicidal behaviors among Bangladeshi university students: Prevalence and risk factors

**DOI:** 10.1371/journal.pone.0262006

**Published:** 2022-01-13

**Authors:** M. Rasheduzzaman, Firoj al-Mamun, Ismail Hosen, Tahmina Akter, Moazzem Hossain, Mark D. Griffiths, Mohammed A. Mamun

**Affiliations:** 1 CHINTA Research Bangladesh, Savar, Dhaka, Bangladesh; 2 Institute of Social Welfare and Research, University of Dhaka, Dhaka, Bangladesh; 3 Department of Public Health and Informatics, Jahangirnagar University, Savar, Dhaka, Bangladesh; 4 Department of Epidemiology, Bangladesh University of Health Sciences, Dhaka, Bangladesh; 5 Institute of Allergy and Clinical Immunology of Bangladesh, Savar, Dhaka, Bangladesh; 6 Psychology Department, Nottingham Trent University, Nottingham, United Kingdom; University of Tripoli, LIBYA

## Abstract

**Background:**

Bangladeshi university students are considered to be highly suicide-prone compared to other populations and cohorts. However, no prior epidemiological studies have assessed the suicidality (i.e., past-year suicidal ideation [SI], lifetime suicide plan [SP], and lifetime suicide attempt [SA]) among Bangladeshi students, including the variables such as past-year stressful life events and family mental health history. This is arguably a major knowledge gap in the country. Therefore, the present study investigated the prevalence and associated risk factors for suicidal behaviors among Bangladeshi university students.

**Methods:**

A cross-sectional study was conducted utilizing a convenience sampling method among a total of 1844 university students between October and November 2019. Data were collected based on the information related to socio-demographics, perceived health-related questions, past-year stressful life events, family mental health history, and suicidal behaviors (i.e., SI, SP, and SA). Chi-square tests and binary logistic regressions were used to analyze the data utilizing SPSS statistical software.

**Results:**

The prevalence of past-year suicidal ideation, lifetime suicide plans, and suicide attempts were 13.4%, 6.0%, and 4.4%, respectively. Females reported significantly higher suicidal behavior than males (i.e., 20.6% vs.10.2% SI; 9% vs. 4.6% SP; and 6.4% vs.3.6% SA). Risk factors for SI were being female, year of academic study, residing in an urban area, using psychoactive substances, experiencing both past year physical and mental illness, experiencing any type of stressful past-year life events, experiencing campus ragging (i.e., senior students abusing, humiliating and/or harassing freshers or more junior students), experiencing family mental illness history, and having family suicide attempt history. SP was associated with several factors including being female, year of academic study, using psychoactive substance, experiencing both past-year physical and mental illness, and experiencing any type of stressful past-year life events. Risk factors for SA were being female, year of academic study, using psychoactive substances, experiencing past-year mental illness, experiencing any type of stressful past-year life events, and having family suicide attempt history.

**Conclusions:**

University students appear to be a vulnerable group for experiencing suicidal behaviors. The present findings warrant rigorous action and early intervention programs such as counseling and other mental health professional services by university authorities. Longitudinal studies are highly recommended involving countrywide representative samples.

## 1 Introduction

Suicidal behaviors can be defined as individuals experiencing repeated thoughts of killing themselves life (suicidal ideation), planning to kill themselves (suicide plan), and actual efforts to kill themselves (suicide attempt), while suicide refers to actually killing themselves. Suicidal behaviors are frequently accompanied by overwhelming hopelessness, depression, or self-destructive behavior (parasuicidal behaviors) [1]. According to a recent meta-analysis, the prevalence of suicidal ideation worldwide is reported to be 10.62% for past-year, 6.14% for lifetime suicide plan, and 3.22% for lifetime suicide attempt [2]. However, suicide has become a global public health problem and accounts for nearly 800,000 deaths among all age groups every year [3]. Of these suicides, 79% of all cases occur in low-income and middle-income countries like Bangladesh [3]. Suicide mostly affects the 15-29-year age group (many of whom are likely to be students) and is the second-highest cause of death after unintentional injury-related deaths from accidents [3].

The present study was carried out in Bangladesh, and a recent Bangladeshi retrospective study reported that individuals aged below 30 years account for almost 61% of the total suicide deaths [4]. Similarly, a few recent retrospective studies using media reports have also explored Bangladeshi students’ suicide vulnerability. For instance, one study reported five student suicides within a ten-day period at the University of Dhaka [5], and another study reported 13 Bangladeshi medical sciences student suicides in a 23-month period [6]. Moreover, another study reported 56 Bangladeshi students’ suicide cases from January 2018 to June 2019 [7].

In Bangladesh, the number of university students has steadily increased over the past few years, but university facilities and subsequent career infrastructure do not meet many students’ needs [8]. Furthermore, there are many problems related to campus and academic life in Bangladesh (i.e., lack of proper accommodation, campus ragging (i.e., senior students abusing, humiliating and/or harassing freshers or more junior students), political violence, poor environment and academic facilities, economic hardship due to living costs) [9, 10]. Along with the aforementioned issues, there are psychological stressors related to the lack of job security and career progression after graduation in Bangladesh [8]. These issues are highly associated with mental health suffering, and recent studies have reported that more than half of Bangladeshi students have mental health issues [9, 11, 12], where similar mental health suffering was noted among the job-seeking graduates [8]. Based on these findings, it is evident that the current Bangladeshi students appear to be at high risk of mental health disorders due to the aforementioned academic and job-related problems. These mental health disorders also contribute to suicide and suicidal behaviors by mediating both distal and proximal suicide risk factors [13–15]. Other risk factors for suicide and suicide-related behaviors include suffering from physical diseases [16–18], stressful life events [14, 19–22], having a family history of mental disorders and suicide [23–26].

A recent meta-analysis claimed that expression of suicidal behaviors (i.e., suicidal ideation) is one of the prominent predictors of suicide completion [27], and successful suicides are often preceded by up to 20 previous attempts suggested by the World Health Organization [3]. But evidence-based data on suicidal behaviors (i.e., suicidal ideation, suicide plans, and suicide attempts) are needed for suicide prevention programs to inform policy-based legislation and public health strategies, public and physician education, and general awareness [3]. Although suicide is one of the preventable public health problems, it has not been effectively addressed in Bangladesh because there is less awareness regarding suicide prevention [7, 22, 28]. Consequently, epidemiological data is much needed for suicide prevention activities in Bangladesh. Therefore, the present study explored suicidal behaviors among Bangladeshi university students and examined associated risk factors (socio-demographics, personal health-related behaviors and traumatic events, and family mental illness and suicide history).

## 2 Methods

### 2.1 Study procedure and participants

A cross-sectional study was conducted among undergraduate students at the University of Dhaka, Bangladesh (mean age = 20.92 years; SD±1.72 years) during October and November 2019. The data were collected through a ‘paper-and-pencil’ survey administered during lectures across all departments of the university by the research team. A convenience sampling technique was used to collect data from participants. Approximately 2,000 students were approached to participate in the survey, with 1897 agreeing to take part (94.6% response rate). Inclusion criteria for the study were (i) being a student of the university and (ii) being present in the class during data collection. Participants were excluded if they were not currently students at the university or were graduate students of the university. After removing the incomplete questionnaires, data from 1844 participants remained for final analysis. Prior to survey completion, study-related issues were introduced, and the research team briefed participants about the whole survey, including the terminology used. The survey took approximately 35 minutes to complete.

### 2.2 Measures

#### 2.2.1 Sociodemographic factors

This survey included questions relating to sociodemographic variables such as age, gender, and whether the participants came from a rural or urban area.

#### 2.2.2 Perceived health-related questions

Self-rated health status, that is, suffering from any type of past-year physical illnesses (e.g., diabetes, asthma, chronic pain, dengue, etc.) and past-year mental health illness (e.g., mood disorders, anxiety disorders, psychotic disorders, trauma-related disorders, etc.) was assessed based on a previous study conducted in Bangladeshi context [29]. Additionally, students were asked if they currently smoked cigarettes and engaged in any other psychoactive substance use (e.g., alcohol, cannabis, illicit drugs, non-medical use of prescription drugs) using a binary response option (i.e., yes/no).

#### 2.2.3 Past-year stressful life events

Past-year stressful life events (i.e., if they had a failure in the examination, if they had relationship complexities, if they were bullied on campus [ragging], if they had family problems, and if they had other problems) were assessed utilizing a binary response ‘yes/no’ response.

#### 2.2.4 Family mental health history

The history of family mental illness (if any of the family members had any mental illness), suicide completion (if any family members had actually committed suicide), and suicide attempt (if any family members attempted suicide) were assessed using a binary response (‘yes/no’) for each of these three variables.

#### 2.2.5 Suicidal behaviors

To assess suicidal behaviors (i.e., suicidal ideation, suicide plans, and suicide attempts), questions used in previous studies were utilized (i.e., binary ‘yes/no’ responses). Participants were asked if they had ever thought about committing suicide during the past year (past-year suicidal ideation; SI), whether such thoughts were persistent across their lifetime, whether they had ever made suicide plans to kill themselves (lifetime suicide plan; SP), and whether they had ever attempted suicide during their lifetime (lifetime suicidal attempt; SA) [30–32].

### 2.3 Ethical considerations

The study followed the medical ethical guidelines of Helsinki Declaration, 1975. The study was reviewed and approved by the ethics board of the Institutional Review Board of the Institute of Allergy and Clinical Immunology of Bangladesh (IACIB), Dhaka, Bangladesh [Reference Number: IRBIACIB/CEC/07201903]. All participants signed an informed consent form prior to participating in the study, and were assured that their data would be anonymous and confidential. They were also informed about the nature, purpose, and procedure of the study, as well as being informed about the right to withdraw their data at any time from the study.

### 2.4 Statistical analysis

This study utilized Statistical Package for Social Science (SPSS) version 22.0 for the data analysis. The analysis included descriptive and inferential statistics such as frequencies, percentages, and means. First-order analysis, including chi-squares and binary logistic regression, also utilized SPSS. All of the variables were added in the unadjusted model (univariate analysis) and then the adjusted model (multivariate analysis) only included the significant variables in the unadjusted model. The unadjusted model was applied for a single predictor and adjusted model was responsible for more than one predictor and where past-year suicidal ideation, lifetime suicide plan, and lifetime suicide attempts were considered as the dependent variables. Odds ratios were used as a measure of risk association, confidence intervals were used as a measure of estimation/precision, and significance levels (*p*<0.05) were used as a measure of statistical significance.

## 3 Results

### 3.1 Characteristics of the participants

The participants’ characteristics are shown in **Table 1**. The sample comprised 70% males, 84.9% came from a village area, 16.7% were cigarette smokers, 3.3% were psychoactive substance users, 10.4% had suffered from physical illnesses in the past year, and 8.4% had experienced mental health psychological suffering. The number of females was less in the present study simply because there was a much smaller proportion of females enrolled at the university. Results also indicated that in the past year, 31.4% had experienced stressful life events, 11.8% had failed examinations, 13.1% had relationship difficulties, 29.2% experienced ragging by other students on campus, 31.0% had experienced family problems, and 8.0% reported experiencing other events (e.g., having personal items stolen [money, smartphone], being in or witnessing a road traffic accident, being humiliated by another person, being beaten up by another person, witnessing others’ injuries and deaths, etc.). Finally, participants reported a history of family mental illness (12.4%), family suicide completion (2.6%) and family suicide attempts (5.4%) (**Table 1**).

**Table 1:** Distribution of the variables with suicidal behaviors.

Variables	Total; *n* (%)	Past-year suicidal ideation (N = 247; 13.4%)			Life-time suicide plans (N = 110; 6.0%)			Life-time suicide attempts (N = 82; 4.4%)		
		Yes; *n* (%)	χ^2^ test value	*p*-value	Yes; *n* (%)	χ^2^ test value	*p*-value	Yes; *n* (%)	χ^2^ test value	*p*-value
**Socio-demographic factors**										
**Gender**										
Female	567; 30.7%	117; 20.6%	36.997	<0.001	51; 9.0%	13.395	<0.001	36; 6.4%	7.018	0.008
Male	1277; 69.3%	130; 10.2%			59; 4.6%			46; 3.6%		
**Year of study**										
4^th^ year	303; 16.5%	65; 21.5%	22.986	0.069	36; 11.9%	23.446	<0.001	29; 9.6%	23.167	<0.001
3^rd^ year	507; 27.6%	50; 9.9%			25; 4.9%			19; 3.8%		
2^nd^ year	519; 28.2%	68; 13.1%			21; 4.0%			20; 3.9%		
1^st^ year	511; 27.8%	63; 12.3%			28; 5.5%			14; 2.7%		
**Permanent residence**										
Rural	1544; 84.9%	192; 12.4%	9.320	<0.001	94; 6.0%	0.169	0.681	72; 4.6%	0.578	0.447
Urban	277; 15.1%	53; 19.1%			15; 5.4%			10; 3.6%		
**Personal health-related variables**										
**Cigarette smoker**										
Yes	308; 16.7%	51; 16.6%	3.296	0.069	32; 10.4%	12.879	<0.001	29; 9.4%	21.425	<0.001
No	1535; 83.3%	195; 12.7%			78; 5.1%			53; 3.5%		
**Psychoactive substance user**										
Yes	60; 3.3%	23; 38.3%	33.249	<0.001	17; 28.3%	55.316	<0.001	17; 28.3%	83.218	<0.001
No	1784; 96.7%	224; 12.6%			93; 5.2%			65; 3.6%		
**Past-year physical health illness**										
Yes	191; 10.4%	66; 34.6%	82.239	<0.001	43; 22.5%	104.012	<0.001	31; 16.2%	69.566	<0.001
No	1653; 89.6%	181; 10.9%			67; 4.1%			51; 3.1%		
**Past-year mental health illness**										
Yes	154; 8.4%	70; 45.5%	148.751	<0.001	59; 38.3%	313.214	<0.001	47; 30.5%	268.472	<0.001
No	1689; 91.6%	177; 10.5%			51; 3.0%			35; 2.1%		
**Past-year stressful life events**										
**Any types of stressful life events during past-year**										
Yes	573; 31.4%	155; 27.1%	134.623	<0.001	86; 15.0%	118.712	<0.001	62; 10.8%	80.228	<0.001
No	1250; 68.6%	89; 7.1%			24; 1.9%			19; 1.5%		
**Examination failure**										
Yes	217; 11.8%	65; 30.0%	58.134	<0.001	33; 15.2%	37.450	<0.001	26; 12.0%	33.136	<0.001
No	1627; 88.2%	182; 11.2%			77; 4.7%			56; 3.4%		
**Relationship difficulties**										
Yes	242; 13.1%	76; 31.4%	77.111	<0.001	53; 21.9%	126.103	<0.001	41; 17.0%	102.933	<0.001
No	1602; 86.9%	171; 10.7%			57; 3.6%			41; 2.6%		
**Campus ragging**										
Yes	120; 6.5%	35; 29.2%	27.523	<0.001	15; 12.5%	9.771	<0.002	6; 5.0%	0.092	0.762
No	1724; 93.5%	212; 12.3%			95; 5.5%			76; 4.4%		
**Family problems**										
Yes	213; 11.6%	66; 31.0%	64.238	<0.001	40; 18.8%	70.492	<0.001	36; 16.9%	87.838	<0.001
No	1631; 88.4%	181; 11.1%			70; 4.3%			46; 2.8%		
**Others**										
Yes	25; 1.4%	2; 8.0%	0.637	0.4257	1; 4.0%	0.175	0.676	1; 4.0%	0.012	0.912
No	1818; 98.6%	245; 13.5%			109; 6.0%			81; 4.5%		
**Family history of psychiatric suffering**										
**Family mental illness history**										
Yes	229; 12.4%	75; 32.8%	84.925	<0.001	42; 18.3%	72.426	<0.001	31; 13.5%	50.651	<0.001
No	1612; 87.6%	171; 10.6%			67; 4.2%			51; 3.2%		
**Family suicide history**										
Yes	48; 2.6%	17; 35.4%	20.603	<0.001	11; 22.9%	25.246	<0.001	6; 12.5%	7.514	0.006
No	1796; 97.4%	230; 12.8%			99; 5.5%			76; 4.2%		
**Family suicide attempt history**										
Yes	99; 5.4%	38; 38.45%	56.314	<0.001	21; 21.2%	43.355	<0.001	18; 18.2%	46.408	<0.001
No	1745; 94.6%	209; 12.0%			89; 5.1%			64; 3.7%		

### 3.2 Prevalence of suicidal behaviors

The present study found that 13.4% of the total participants had past-year suicidal ideation (SI), whereas 6.0% reported having made lifetime suicide plans (SP), and 4.4% had at least one-lifetime suicide attempt (SA) (**Table 1**).

### 3.3 Association between socio-demographics and suicidal behaviors

Results demonstrated that in relation to gender, females had higher rate of experiencing suicidal behaviors compared to males for SI (20.6% vs. 10.2%; χ^2^ = 36.997, *p*<0.001), SP (9% vs. 4.6%; χ^2^ = 13.395, *p*<0.001) and SA (6.4% vs. 3.6%; χ^2^ = 7.018, *p* = 0.008). Fourth-year students had significantly higher SP (11.9% vs. 5.5%, 4% and 4.9%; *p*<0.001) and SA (9.6% vs. 2.7%, 3.9%, and 3.8%; *p*<0.001) compared to first-year, second-year, and third-year year students respectively. Students from urban areas had higher SI compared to rural areas students (19.1% vs.12.4%; *p*<0.001; **Table 1**).

### 3.4 Association between health-related variables and suicidal behaviors

Results indicated that cigarette smoking was not associated with SI, but was significantly associated with SP (10.4% vs. 5.1%; χ^2^ = 12.879, *p*<0.001) and SA (9.4% vs. 3.5%; χ^2^ = 21.425, *p*<0.001). Psychoactive substance users reported a higher significant rate of all suicidal behaviors compared to non-users. Similarly, participants with a past-year health suffering (both physical and psychological) reported significantly higher levels of all types of suicidal behaviors than those who had no health suffering (**Table 1**).

### 3.5 Association between past-year stressful life events and suicidal behaviors

Participants with a history of any type of past-year stressful life event (compared to those that did not) had significantly higher levels of SI (27.1% vs. 7.1%; χ^2^ = 134.623, *p*<0.001), SP (15.0% vs. 1.9%; χ^2^ = 118.712, *p*<0.001) and SA (10.8% vs. 1.5%; χ^2^ = 80.228, *p*<0.001). Similarly, past-year stressful life events were significantly associated with suicidal behaviors. This including examination failure (SI: χ^2^ = 58.134, *p*<0.001; SP: χ^2^ = 37.450, *p*<0.001; and SA: χ^2^ = 33.136, *p*<0.001), relationship difficulties (SI: χ^2^ = 77.111, *p*<0.001; SP: χ^2^ = 126.103, *p*<0.001; and SA: χ^2^ = 102.933, *p*<0.001), family problems (SI: χ^2^ = 64.238, *p*<0.001; SP: χ^2^ = 70.492; and *p*<0.001; and SA: χ^2^ = 87.838, *p*<0.001) and being ragged by other students on campus (SI: χ^2^ = 27.523, *p*<0.001; and SP: χ^2^ = 9.771, *p*<0.001 –although it was not associated with SA: χ^2^ = 0.092, *p* = 0.762) (**Table 1**).

### 3.6 Association between family mental health history and suicidal behaviors

Participants with a family history of psychiatric illness (compared to those who did not) had significantly higher levels of SI (32.8% vs. 10.6%; χ^2^ = 84.925, *p*<0.001), SP (18.3% vs. 4.2%; χ^2^ = 72.426, *p*<0.001) and SA (13.5% vs. 3.2%; χ^2^ = 50.651, *p*<0.001). Similarly, participants with a suicide-related family history also had higher levels of all types of suicide behaviors compared to those that did not [i.e., suicide completion (χ^2^ = 20.603, *p*<0.001; χ^2^ = 25.246, *p*<0.001; and χ^2^ = 7.514, *p* = 0.006 for SI, SP and SA respectively) and suicide attempt (χ^2^ = 56.314, *p*<0.001; χ^2^ = 43.355, *p*<0.001; and χ^2^ = 46.408, *p*<0.001 for SI, SP and SA respectively)] (**Table 1**).

### 3.7 Risk factors for suicidal ideation

**Table 2** shows the risk factors for suicidal ideation utilizing multivariate analysis (Nagelkerke’s R^2^ = 0.259). The significant predictors were gender (using male as reference; AOR = 2.257, 95% CI = 1.60–3.17), year of academic study (using first-year as reference; AOR = 0.53, 95% CI = 0.34–0.83), residence (using living in an urban area as reference, AOR = 0.61, 95% CI = 0.42–0.90), past-year physical illness (using no physical illness as reference, AOR- 1.80, 95% CI = 1.19–2.73), past-year mental illness (using no mental illness as reference, AOR = 2.69, 95% CI = 1.73–4.22), any type of past-year stressful life events (using no past-year stressful life events as reference, AOR = 2.20, 95% CI = 1.45–3.34), family mental illness history (using no family mental illness history as reference, AOR = 1.56, 95% CI = 1.05–2.33), family suicide attempt history (using no family suicide attempt as reference, AOR = 2.07, 95% CI = 1.22–3.49) (**Table 2**).

**Table 2:** Logistic regression analysis of the variables associated with suicidal ideation.

Variables	Unadjusted model			Adjusted model (-2 Log likelihood = 1137.861; Nagelkerke’s R^2 =^ 0.259)		
	Odds ratio (OR)	95% Confidence Interval (CI)	*p*-value	Adjusted odds ratio (AOR)	95% Confidence Interval (CI)	*p*-value
**Gender**						
Female	2.29	1.74–3.01	<0.001	2.25	1.60–3.17	<0.001
**Male**	Reference			Reference		
**Year of study**						
1^st^ year	0.51	0.35–0.75	<0.001	0.53	0.34–0.83	0.014
2^nd^ year	0.55	0.38–0.80		0.63	0.41–0.97	
3^rd^ year	0.40	0.26–0.59		0.51	0.32–0.81	
4^th^ year	Reference			Reference		
**Permanent residence**						
Rural	0.59	0.42–0.83	0.002	0.61	0.42–0.90	0.014
Urban	Reference			Reference		
**Cigarette smoker**						
Yes	1.36	0.97–1.90	0.070	1.35	0.85–2.15	0.191
No	Reference			Reference		
**Psychoactive substance user**						
Yes	4.32	2.52–7.42	<0.001	2.13	1.00–4.55	0.049
No	Reference			Reference		
**Past-year physical illness**						
Yes	4.29	3.06–6.00	<0.001	1.80	1.19–2.73	0.005
No	Reference			Reference		
**Past-year mental illness**						
Yes	7.11	5.00–10.13	<0.001	2.69	1.72–4.22	<0.001
No	Reference			Reference		
**Any type of stressful life event during past year**						
Yes	4.83	3.64–6.42	<0.001	2.20	1.45–3.34	<0.001
No	Reference			Reference		
**Examination failure**						
Yes	3.39	2.44–4.71	<0.001	1.34	0.89–2.01	0.153
No	Reference			Reference		
**Relationship difficulties**						
Yes	3.83	2.79–5.24	<0.001	1.18	0.77–1.81	0.444
No	Reference			Reference		
**Campus ragging**						
Yes	2.93	1.93–4.46	<0.001	1.71	1.01–2.89	0.044
No	Reference			Reference		
**Family problems**						
Yes	3.59	2.58–4.99	<0.001	1.21	0.78–1.86	0.381
No	Reference			Reference		
**Other problems**						
Yes	0.55	0.13–2.38	0.432	0.79	0.17–3.55	0.761
No	Reference			Reference		
**Family mental illness history**						
Yes	4.10	2.98–5.64	<0.001	1.56	1.05–2.33	0.027
No	Reference			Reference		
**Family suicide history**						
Yes	3.73	2.03–6.85	<0.001	1.32	0.63–2.75	0.456
No	Reference			Reference		
**Family suicide attempt history**						
Yes	4.57	2.97–7.03	<0.001	2.07	1.22–3.49	0.006
No	Reference			Reference		

### 3.8 Risk factors for suicide planning

**Table 3** shows the risk factors for suicide planning utilizing multivariate analysis (Nagelkerke’s R^2^ = 0.384). The significant predictors were gender (using male as reference, AOR = 2.03, 95% CI = 1.21–3.42), year of academic study (using first-year as reference, AOR = 0.52, 95% CI = 0.27–0.98), psychoactive substance user (using no psychoactive substance use as reference, AOR = 2.74, 95% CI = 1.07–7.02), past-year physical illness (using no past-year physical illness as reference, AOR = 2.09, 95% CI = 1.22–3.58), past-year mental illness (using no past-year mental illness as reference, AOR = 7.74, 95% CI = 4.50–13.32), any type of stressful past-year life events (using no type of stressful past-year events as reference, AOR = 3.03, 95% CI = 1.62–5.68) (**Table 2**).

**Table 3:** Logistic regression analysis of the variables associated with suicide plans.

Variables	Unadjusted model			Adjusted model (-2 Log likelihood = 545.245; Nagelkerke’s R^2 =^ 0.384)		
	Odds ratio (OR)	95% Confidence Interval (CI)	*p*-value	Adjusted odds ratio (AOR)	95% Confidence Interval (CI)	*p*-value
**Gender**						
Female	2.04	1.38–3.00	<0.001	2.03	1.21–3.42	0.007
Male	Reference			Reference		
**Year of study**						
1^st^ year	0.43	0.25–0.72	<0.001	0.52	0.27–0.98	0.012
2^nd^ year	0.31	0.17–0.54		0.32	0.16–0.64	
3^rd^ year	0.38	0.22–0.65		0.51	0.27–0.99	
4^th^ year	Reference			Reference		
**Permanent Residence**						
Rural	1.12	0.64–1.97	0.681	1.31	0.66–2.62	0.430
Urban	Reference			Reference		
**Cigarette smoker**						
Yes	2.16	1.40–3.33	<0.001	1.59	0.82–3.09	0.167
No	Reference			Reference		
**Psychoactive substance user**						
Yes	7.18	3.94–13.08	<0.001	2.74	1.07–7.02	0.035
No	Reference			Reference		
**Past-year physical illness**						
Yes	6.87	4.52–10.45	<0.001	2.09	1.22–3.58	0.007
No	Reference			Reference		
**Past-year mental illness**						
Yes	19.94	13.00–30.60	<0.001	7.74	4.50–13.32	<0.001
No	Reference			Reference		
**Any type of stressful life events during past-year**						
Yes	9.02	5.66–14.35	<0.001	3.03	1.62–5.68	<0.001
No	Reference			Reference		
**Examination failure**						
Yes	3.61	2.33–5.58	<0.001	1.11	0.63–1.94	0.711
No	Reference			Reference		
**Relationship difficulties**						
Yes	7.60	5.07–11.37	<0.001	1.51	0.86–2.65	0.151
No	Reference			Reference		
**Campus ragging**						
Yes	2.45	1.37–4.37	0.002	0.96	0.45–2.06	0.935
No	Reference			Reference		
**Family problems**						
Yes	5.15	3.39–7.84	<0.001	1.19	0.68–2.09	0.540
No	Reference			Reference		
**Other problems**						
Yes	0.65	0.08–4.87	0.678	0.99	0.11–8.44	0.997
No	Reference			Reference		
**Family mental illness history**						
Yes	5.17	3.42–7.83	<0.001	1.39	0.79–2.45	0.252
No	Reference			Reference		
**Family suicide history**						
Yes	5.09	2.52–10.29	<0.001	1.41	0.57–3.47	0.455
No	Reference			Reference		
**Family suicide attempt history**						
Yes	5.01	2.95–8.48	<0.001	1.75	0.88–3.48	0.110
No	Reference			Reference		

### 3.9 Risk factors for suicide attempts

**Table 4** shows the risk factors for suicide attempts utilizing multivariate analysis (Nagelkerke’s R^2 =^ 0.379). The significant risk factors were gender (using male as reference, AOR = 2.02, 95% CI = 1.11–3.67), year of academic study (using first-year as reference, AOR = 0.34, 95% CI = 0.15–0.74), psychoactive substance user (using no psychoactive substance use as reference, AOR = 3.62, 95% CI = 1.33–9.86), past-year mental illness (using no past-year substance use as reference, AOR = 8.71, 95% CI = 4.72–16.07), any type of past-year stressful life events (using no past-year stressful life events as reference, AOR = 2.15, 95% CI = 1.01–4.43), and family suicide attempt history (using no family suicide attempt history as reference, AOR = 2.38, 95% CI = 1.13–5.03) (Table 4).

**Table 4:** Logistic regression analysis of the variables associated with suicide attempt.

Variables	Unadjusted model			Adjusted model (-2 Log likelihood = 438.198; Nagelkerke’s R^2 =^ 0.379)		
	Odds ratio (OR)	95% Confidence Interval (CI)	*p*-value	Adjusted odds ratio (AOR)	95% Confidence Interval (CI)	*p*-value
**Gender**						
Female	1.81	1.16–2.84	0.009	2.02	1.11–3.67	0.020
Male	Reference			Reference		
**Year of study**						
1^st^ year	0.26	0.13–0.51	<0.001	0.34	0.15–0.74	0.035
2^nd^ year	0.37	0.21–0.68		0.48	0.23–0.98	
3^rd^ year	0.36	0.20–0.67		0.50	0.24–1.03	
4^th^ year	Reference			Reference		
**Permanent Residence**						
Rural	1.29	0.66–2.54	0.448	1.85	0.80–4.28	0.147
Urban	Reference			Reference		
**Cigarette smoker**						
Yes	2.90	1.81–4.64	<0.001	1.86	0.89–3.92	0.099
No	Reference			Reference		
**Psychoactive substance user**						
Yes	10.44	5.65–19.30	<0.001	3.62	1.33–9.86	0.012
No	Reference			Reference		
**Past-year physical illness**						
Yes	6.08	3.78–9.78	<0.001	1.46	0.78–2.72	0.232
No	Reference			Reference		
**Past-year mental illness**						
Yes	20.74	12.84–33.50	<0.001	8.71	4.72–16.07	<0.001
No	Reference			Reference		
**Any type of stressful life events during past-year**						
Yes	7.87	4.66–13.30	<0.001	2.15	1.05–4.43	0.036
No	Reference			Reference		
**Examination failure**						
Yes	3.83	2.35–6.25	<0.001	1.30	0.70–2.44	0.400
No	Reference			Reference		
**Relationship difficulties**						
Yes	7.80	4.94–12.32	<0.001	1.67	0.87–3.21	0.121
No	Reference			Reference		
**Campus ragging**						
Yes	1.14	0.48–2.67	0.762	0.43	0.15–1.18	0.102
No	Reference			Reference		
**Family problems**						
Yes	7.00	4.40–11.12	<0.001	1.62	0.88–2.99	0.121
No	Reference			Reference		
**Other problems**						
Yes	0.89	0.11–6.68	0.913	1.47	0.17–12.73	0.725
No	Reference			Reference		
**Family mental illness history**						
Yes	4.78	2.99–7.66	<0.001	1.24	0.64–2.37	0.517
No	Reference			Reference		
**Family suicide history**						
Yes	3.23	1.33–7.83	0.009	0.69	0.23–2.08	0.521
No	Reference			Reference		
**Family suicide attempt history**						
Yes	5.83	3.30–10.29	<0.001	2.38	1.13–5.03	0.022
No	Reference			Reference		

## 4 Discussion

In the present study, findings indicated that the prevalence rate among Bangladshi students for (i) past-year suicidal ideation (SI) was 13.4%, (ii) lifetime suicide plans (SP) was 6.0%, and (iii) lifetime suicide attempt (SA) was 4.4% respectively. In other Bangladeshi studies, the rate of past-year suicidality among university students was reported to be 28.5% in a multi-institutional study [11], 12.4% among dental students [10], and 17.7% among university entrance test exam students [33]. Compared to prior Bangladeshi studies, it is evident that the reported suicidal ideation in the present study appears to be lower.

A study of 19 colleges comprising 13,984 first-year students across eight countries (i.e., Australia, Belgium, Germany, Mexico, Northern Ireland, South Africa, Spain, and the United States) reported prevalence rates of 17.2% for past-year SI prevalence, 17.5% for lifetime SP, and 4.3% for lifetime SA [34]. Another study examining adolescents from 32 low-income and middle-income countries, reported a pooled past-year SI prevalence rate of 12.2% for males (11.7%-12.7%) and 16.2% for females (15.6%-16.7%) [30], compared to a past-year prevalence rate of 18.2% among Ghanaian high school students (N = 1,984 [31]). However, a recent meta-analysis among 36 studies comprising college students (N = 634,662 students: 15 undergraduate samples, four graduate samples, 11 mixed undergraduate/graduate samples, and six not reported) estimated prevalence rates of 10.62% for past-year SI (9.10% to 12.25%), 6.14% for lifetime SP (4.78% to 7.75%) and 3.22% for lifetime SA (2.16% to 4.46%) [2]. Based on the aforementioned suicidal behaviors prevalence rates, it can be concluded that the present sample had a higher prevalence of suicidal behaviors for SI (13.4% vs. 10.62%) and SA (4.4% vs. 3.22%), and an equivalent prevalence rate for SP (6.0% vs. 6.14%). These higher rates may be particularly due to the university itself because previous research in Bangladesh examining actual suicides (rather than suicidal behaviors more generally) at the same university as the present study (i.e., University of Dhaka) reported five suicidality cases within a 10-day period [5].

Globally, gender differences on suicidal death and suicidal behaviors have been consistent (i.e., the female suicide rate is lower than males, but they experience a higher prevalence of suicide-related behaviors–such as SA–compared to males) [35]. Compared to findings globally (i.e., more SA among females) and Bangladeshi suicide trends (i.e., more suicides among females), the present study’s findings are consistent (i.e., females had higher prevalence rates among all types of suicidal behavior). In addition, depending on the reasons for suicide, the difference between males and females has been found to be higher due to economic problems, relationship problems, and educational failure [36]. Studies have also reported that relationship complexities are the primary cause of suicide among females, whereas economic concerns and illness are the major causes of suicide among males [36, 37]. Moreover, other biological and/or psychological factors, including coping style, impulsivity, and personality, may influence gender differences in suicidal behaviors.

It should also be noted that the adjusted model in the present study provides a more accurate depiction of the risk factors associated with suicidal behaviors than the unadjusted model. Moreover, the present study found higher prevalence rates of all suicidal behaviors among psychoactive substance users (i.e., alcohol, cannabis, illicit drugs, non-medical use of prescription drugs), and cigarette smoking was significantly associated with both suicide planning and suicide attempts (but not suicidal ideation). Previous research indicates that substance abuse can have a wide range of direct and indirect effects on both physical and mental health. As reported in a recent systematic review [38], there are significant associations between all types of substance use and suicidal behaviors. These effects often depend upon the drug specification, amount of use, frequency of use, personal health capabilities, and other factors. However, the present study did not consider these factors [38]. Therefore, further studies are needed to examine these specific relationships and factors between substance use and suicidality.

Strong relationships between physical illnesses and extreme mental health conditions (i.e., suicide and suicidality) are well-established [16]. Physical illnesses (e.g., high blood pressure, heart attacks, strokes, arthritis, chronic headaches, other chronic pain, respiratory conditions and bronchial asthma, diabetes, arthritis, hypothyroidism, etc.) can predispose individuals to mental illnesses by mediating abnormal and imbalanced secretions of neurotransmitters (e.g., serotonin, dopamine, norepinephrine, etc.) that make individuals more suicide-prone (even in the absence of any mental disorders; [16]), have also been reported in the Bangladeshi literature [18]. Individuals with mental disorders (with or without physical illnesses) are also at high risk of suicide-related behaviors and have been reported globally [16, 27]. In Bangladesh, recent retrospective studies reported that up to 60% of individuals with SI experience depression and other disorders such as schizophrenia, bipolar disorders, obsessive-compulsive disorder, generalized anxiety disorder, personality disorders, anxiety disorder, panic disorder, and conversion disorder [18, 39, 40]. Consistent with the prior studies, this study found a higher risk of suicidal behaviors of these participants with either mental health problems or physical illnesses.

Negative and traumatic life experiences such as criminal victimization, interpersonal violence (e.g., being raped, sexually molested, physically assaulted, physically abused as a child, seriously neglected as a child, threatened with a weapon, held captive or kidnapped), non-interpersonal violence (e.g., suffering great shock, life-threatening accidents, fire/flood/natural disasters, and witnessing bad injuries/deaths), domestic violence, childhood abuse and neglect, torture, sexual traumatization, natural disasters, and holocausts, are highly associated with suicidal behaviors and suicide contribution [21, 23], also the findings of the present study support this.

Several possible pathways between exposure to traumatic events have been suggested, including the mediating role of post-traumatic stress disorder (PTSD) symptoms, depression, psychiatric comorbidity, and dissociation, as well as the impact upon personality and cognitive development [19–21]. As consistent with the aforementioned literature, Bangladeshi studies also suggest that students’ negative events (i.e., lack of proper accommodation, campus ragging, and political violence, etc.) mediate common psychological problems such as depression, anxiety, and stress [9], and these disorders contribute proximal suicide risk factors [22]. In the present study, stressful life-events (e.g., examination failure, relationship difficulties, campus ragging, family problems, etc.) were highly associated with all suicidal behaviors, although campus ragging did was not a risk factor for SA. In addition, experiencing a self-reported physical and mental illness were significantly associated with SI, SP and SA (except physical co-morbidities) in the present study. This finding can be explained by the relationship between physical illness, mental illness, and suicidal behavior where physical co-morbidities can trigger psychiatric disorder alongside feelings of hopelessness or helplessness, a dramatic change in personality or appearance and/or irrational or bizarre behaviour. It has also been reported that psychiatric disorders are estimated to be responsible for a large proportion of suicides [41, 42].

This study also found the importance of the family history of mental illness, and suicide and suicidal behaviors in the association of all suicidal behaviors. As reported previously, both fatal and non-fatal suicidal behaviors of offspring are consistently associated with a history of affective and mood disorders, substance abuse, internal family conflicts, inappropriate parent-child relationships, history of suicide completion, and suicide attempts within the family [23–26]. Previous Bangladeshi findings are the same (e.g., 16.5% of individuals with SI had a family SA history [43]).

The present study has a number of limitations including (i) it being a cross-sectional study, (ii) assessing mental health illness and physical health illness using self-report, and (iii) a limited number of variables being examined and the omission of potentially important variables (e.g., family income, relationship status, childhood maltreatment, etc.). Moreover, assessing only a single university in Bangladesh limits the generalizability of the findings for other universities inside or outside of the country. Therefore, future (preferably longitudinal) research using countrywide representative student samples is needed to establish causal pathways between the variables examined in the present study. Despite these limitations, the study presented novel data concerning students’ suicidal behaviors using a relatively large sample which will hopefully facilitate suicide prevention initiatives to be implemented by university authorities as well as further studies in the country.

## 5 Concluding remarks

Based on the present research (and elsewhere [9, 10]), campus-related issues such as ragging (among freshers) and examination failure (among final-year students) are prominent problems that should also be taken into account when developing suicide prevention programs on campus. However, other issues such as relationship complexities, family problems, and psychoactive substance abuse also require consideration in such programs. Additionally, providing a student-friendly campus environment with appropriate psychological support (i.e., gatekeeper training, mental health support programs, etc.) is recommended based on the present findings.

## Supporting information

S1 Data (SAV)
